# Partner influences on young women’s risky drug and sexual behavior

**DOI:** 10.1186/s12978-018-0598-0

**Published:** 2018-09-15

**Authors:** Miriam Clark, Rohanna Buchanan, Rachel Kovensky, Leslie D. Leve

**Affiliations:** 1 Oregon Social Learning Center, 10 Shelton McMurphey Blvd, Eugene, OR 97401 USA; 2 Prevention Science Institute, 6217 University of Oregon, Eugene, OR 97403 USA

**Keywords:** Drug use, Health-risking sexual behavior, Adolescent experiences, Partner influence, Qualitative interview

## Abstract

**Background:**

Adolescent girls with a history of maltreatment are at heightened risk for health-risking behaviors, including unsafe sexual behaviors and drug use. However, few studies have examined the views of this population in regard to sexual partner choice, sexual behaviors, and decisions to use drugs with sexual partners.

**Methods:**

We conducted 15 semistructured, open-ended qualitative interviews with young women ages 18–24 with a history of maltreatment and asked them to reflect on their experiences as adolescents. We used the constant comparison method to group the qualitative coded data into themes.

**Results:**

Analysis of the interviews suggested that adolescent girls with maltreatment histories often report that they chose partners who are promotive of risky drug and sexual behavior. The interviews also provided insight into why this population is likely to use drugs with their partner and why they might be hesitant to talk about or practice safe sex with their partner.

**Conclusion:**

The young women’s feedback highlighted five areas where adolescent girls with maltreatment histories could benefit: (a) provision of information about partner characteristics that are promotive of both risky behavior and those that are linked to healthy relationships, (b) provision of information about how one’s partner can influence one’s own drug use, (c) practice talking about safe sex with partners, (d) provision of information about safe sex practices and the risks associated with unsafe sex, and (e) provision of information about the risks associated with drug use and unsafe sexual behavior to adolescent boys.

## Plain english summary

In our study we talked to young women who had been maltreated as adolescents and were subsequently placed in out-of-home treatment (foster care or treatment facilities). This population is at heightened risk for unsafe sexual behavior and drug use. We asked the young women how their romantic relationships influenced their sexual behavior and drug use when they were adolescents. The women told us how the partners they chose as adolescents often encouraged unsafe sex and drug use, and that they were often hesitant to talk to their partners about these issues. Through these interviews, we received insight into topics for practitioners to focus on when working with adolescents. The women’s stories highlight that adolescent girls could benefit from knowing about partner characteristics that are likely to lead to risky sex and drug use, and learning more about how to talk about and practice safe sex with their partners.

## Background

Adolescents with a history of maltreatment are at heightened risk for engaging in multiple forms of health-risking behaviors, including unsafe sexual behaviors and drug use [1–6]. Unsafe sexual behaviors and drug use (via needle injection) are highly correlated with sexually transmitted diseases, including HIV/AIDS and other negative health consequences [https://www.cdc.gov/pwud/]. Young women in this population often bear more health burdens than do their male counterparts because of specific health consequences, including unwanted pregnancies, ectopic pregnancies and infertility [7]. In addition, adolescent girls are also more likely than adolescent boys to become addicted to drugs [8, 9], experience intimate partner violence [10], and contract sexually transmitted infections (STI’s) [11].

Romantic partners (defined as dating or sexual partners) have a significant impact on the sexual behaviors and drug use of adolescent girls with maltreatment histories [12, 13]. Despite high rates of romantic relationships in this population [14], very few studies have focused on the role of partners on adolescent girls’ drug use and sexual behaviors. To help fill this gap, we sought to learn retrospectively about the partner experiences of adolescent girls with a history of maltreatment. Using a semistructured qualitative interview format, we asked young women to reflect on their experiences as adolescents and discuss the relationships they had with their partners. We asked questions about how they chose their partners and the impact their partners had on their sexual behavior and drug use. We also explored reasons why adolescent girls with a history of maltreatment used drugs and/or engaged in unsafe sexual behaviors with their partners.

### Health-risking behaviors in adolescent girls

Problem behavior theory [15] suggests that young people who engage in one type of risky behavior in youth (such as drug use, early or risky sexual behavior, and delinquent behaviors) are more likely to also engage in other types of risky behavior. Findings from longitudinal studies of adolescents and young adults have confirmed that young people who engage in risky or problem behavior often engage in multiple types of such behavior [15, 16]. This theory guided the current study questions and our qualitative interview process to jointly focus on a range of health-risking behaviors, including drug use and sexual behavior.

Health-risking behaviors, such as unprotected sex and drug use, often emerge in adolescence and peak during young adulthood [17], resulting in immediate developmental consequences and deleterious health outcomes that can persist across the lifespan. According to the Centers for Disease Control and Prevention [18], individuals between ages 15 and 24 comprise half of the nearly 20 million newly diagnosed sexually transmitted infections (STIs) in the United States every year. Further, more than 1.2 million people are currently living with HIV in the United States, and approximately 19% of those who are newly diagnosed with HIV are women. Of women diagnosed, 87% have contracted HIV from heterosexual contact and 13% from needle injection [19].

The National Center on Addiction and Substance Abuse [20] reports that 76% of high school students have used one or more addictive drugs. Although prevalence of STIs and drug use is relatively evenly distributed among adolescent boys and girls [11, 18, 21], adolescent girls often bear disproportionate health consequences (e.g. life-threatening ectopic pregnancy, infertility, unwanted pregnancy) and are biologically predisposed to be more susceptible to certain STI’s [11]. In addition, adolescent girls have a heightened risk for sexual violence [10] and addiction once drug use is initiated [8, 9]. Adolescent and adult women with a history of childhood maltreatment are a subgroup at particularly high risk for participating in unsafe sexual behavior [2, 4–6]. Using a large nationally representative sample of young adult women, Hahm, Lee, Ozonoff, and Van Wert [22] found a significant cumulative relationship between increased exposure to multiple types of maltreatment and increased risk for STI diagnosis and early sexual contact. Adults with a history of child maltreatment are also more likely to engage in unprotected sex with casual partners [2].

Adolescent girls and adult women with a maltreatment history are also more likely to have problems with drug use than their non-maltreated counterparts. For example, Fettes, Aarons, and Green [3] compared two nationally representative samples of adolescents and found that adolescents with a maltreatment history were significantly more at risk for drug use involvement than were those without a maltreatment history. These findings are consistent with research that has documented higher rates of drug use disorders among adolescents with a maltreatment history [1]. Drug use and unsafe sexual behavior are linked, with adolescents and adults with a history of maltreatment being more likely to have sex while drinking alcohol or using drugs, which often leads to unprotected, casual sex [4–6]. Although the elevated risks and serious consequences of unsafe sexual behavior and drug use are fairly well established among adolescent girls with a history of maltreatment, relatively little is known about pathways to these health-risking behaviors among this population [23] and about pathways to healthy sexual behavior in this population [24].

The United Nations Educational, Scientific and Cultural Organization (UNESCO) recently published a resource detailing the core elements of sexual education programs [25]. The UNESCO resource advocates teaching young people—through skill based learning—how to recognize relationship power dynamics, how to seek help and support, and how to negotiate safe sex practices [25]. Findings from a review of programs to promote positive youth development (PYD) suggests that safer sexual behavior tends to be correlated with other positive psychosocial behaviors (such as sports, academic activities, and goal setting) and that effective PYD programs that target one of these often affect the others [24]. In addition, the findings from this review of PYD programs suggest that including training and activities related to goal setting, social skills, and engagement in positive social activities can enhance sexual education programs [24].

### Partner influences on drug use and sexual behavior

One potential pathway linking drug use and sexual behaviors among adolescent girls and young women with a maltreatment history is the influence of their romantic partners. A large body of evidence suggests that child maltreatment is associated with problematic romantic relationships later in life [26]. Specifically, McLeod, Fergusson, & Horwood [27] found a significant association between childhood maltreatment and elevated negative partner relationships, interpersonal violence-related victimization and perpetration, and choosing partners with social problems related to alcohol use and illegal behavior in their 30-year longitudinal study of a large New Zealand birth cohort. Adolescent girls who select partners who are involved in antisocial behavior, such as drug use and delinquency, appear to be at particular risk for engaging in these behaviors themselves. For instance, Rhoades, Leve, Harold, Kim, & Chamberlain [28] found that among a sample of women with juvenile justice and child welfare history, drug use was significantly associated with their partners’ use. Silverman, Raj, Mucci, & Hathaway [13] found that adolescent girls who were in violence-prone dating relationships were significantly more likely to use substances (e.g., alcohol, tobacco, cocaine), engage in risky sexual behavior (e.g., early sexual debut, multiple partnering), and seriously consider or attempt suicide.

Additionally, research shows adolescent girls are more likely to participate in risky sexual behavior when partners are older than them [29, 30], when they are driven by a sense of need to be in a relationship [31], or when they rely heavily on their partner for emotional or financial support [32]. Conversely, self-regulation (ability to regulate behavior, attention, and emotions) is associated with lower sexual risk taking in adolescents, as is parental control and lower levels of negative peer pressure [33].

Despite the known associations between childhood maltreatment and young women’s sexual behavior, there is very little research on the association between childhood maltreatment and young women’s feelings about safe sex. In one of the few studies on this topic, women filled out a childhood sexual abuse questionnaire and then read a sexual scenario involving either alcohol or no alcohol. Results indicated that childhood sexual abuse was a predictor of the women’s perception of the partner’s motives and intentions, which was then a predictor of the likelihood she would report she would engage in unprotected sex [34]. The authors suggested that women with maltreatment histories might make decisions in different ways than would their non-maltreated peers. Although not specific to a sample of participants with maltreatment histories, a quantitative study examining women living in high poverty, crime, and drug use environments reported that the women’s attitudes about condom use are quite variable [35]. For example, although the majority of the women said that condoms did *not* reduce sexual pleasure, a quarter of the women still listed reduction in sexual pleasure as a reason they did not use condoms. Because of the variability of women’s responses, further research is needed to more fully understand women’s opinions of condom use and how their opinions are influenced by their partner.

Although evidence points to the influential role partners play in determining whether or not adolescent girls engage in drug use and sexual behaviors, little is known about the mechanisms underlying partner choice or the degree to which specific partner traits predict health-risking behaviors in the relationship [12]. To fill this gap in the literature, Rhule-Louie & McMahon [36] suggest that qualitative interviews could be particularly effective for clarifying the factors that underlie partner selection and for informing interventions to influence adolescent partner selection.

### This study

We conducted 15 semistructured, open-ended qualitative interviews with young adult women with a documented history of maltreatment that had resulted in placement in foster care or other out-of-home care setting during childhood and/or adolescence. The interviews focused on the adolescents’ experiences with regard to sexual partner choice decisions to engage in safe or unsafe sexual behavior, and decisions to use alcohol and drugs with sexual partners. Qualitative interviews and analyses were guided by two research questions: (1) How do adolescent girls who have experienced maltreatment choose romantic partners? (2) What influence do romantic partners have on adolescent drug use and sexual behavior in this population? Our goal for the current study was to learn about malleable relationship-based processes that could be targeted in future preventive interventions with high-risk adolescent and young adult female populations, including those who have experienced maltreatment.

## Methods

### Participants

Participants were 15 women with a history of maltreatment. Participants were selected based on having had a childhood history of maltreatment (ascertained via child welfare case worker report) and having engaged in sexual behavior as an adolescent (ascertained via responses to a questionnaire administered when the participants were adolescents). Ethics approval for the study was granted by the University of Oregon Institutional Review Board (IRB; 10,312,013.040). For the current study, all of the young women participated in an informed consent procedure that was individually administered.

Study participants were women ages 18 to 24 (*M* = 20.93, *SD* = 2.09) at the time of the interview. They identified as White or Caucasian (60%), American Indian/Alaska Native (7%), or more than one race (33%). Overall, 20% identified as Hispanic/Latina. One participant reported that she was married, 6 were living with a partner and unmarried, and 8 were dating or seeing someone but not living together or married. Participants reported a range of educational attainment, including having completed the eighth grade (*n* = 1), completed some high school (*n* = 4), received a GED (*n* = 3), received a high school diploma (*n* = 5), and completed some college (*n* = 2). The sample was generally unemployed and low income, with only 4 women employed for pay and a median annual income ranging from $5000 to $9999.

### Measures

#### Interview guide

We used an individually administered, semistructured, and open-ended qualitative interview format and conducted one interview with each participant. Interviews were scheduled for 2 h in duration though the average length was 102 min and ranged from 66 to 136 min. The interview guide included broad, general questions designed to prompt respondents to think retrospectively about their adolescent years and to solicit information about the topics most important to them. The initial interview questions focused on the women’s prior treatment and intervention experiences (see Clark, Buchanan, & Leve, 2018 [37] for details). To gain a broad understanding of how adolescent girls choose romantic partners and partners’ influence on drug use and sexual behavior, we next asked general or hypothetical questions to create a pressure-free environment and provide an opportunity for respondents to give concrete examples. Example questions included, “What do you think adolescents look for in a partner?” and, “We know from previous research studies that when a girl’s partner uses drugs, she’s more likely to use drugs too. Why do you think this might be?” Interviewers were trained in techniques to elicit information from participants, remain neutral to a range of responses, and move the discussion through the semistructured format.

#### Demographic survey

Immediately prior to the qualitative interview, participants were given a demographic survey that included items related to their age, race/ethnicity, current relationship status, highest education level completed, current occupation and employment status, and current gross annual household income.

### Analysis

All interviews were audio recorded and transcribed verbatim. The transcriptions were then reviewed by the interviewer for accuracy and completeness. Participants and other individuals identified in the transcripts were assigned an identification number to protect confidentiality, increase cross-group comparisons, and reduce subjectivity in the analyses [38]. After reviewing all the interviews, the authors met and discussed broad themes identified across all transcripts. Using NVIVO software [39], the first author completed an initial coding of the broad themes for each interview. Next, a codebook emerged as the first author coded sections of text (ranging in size from short phrases to long discussions) by hand into more specific emergent subthemes and then compared the coded content across each interview. The constant comparison method [40] was used to group codes into themes. All authors met to discuss findings related to the broad themes and subthemes and to identify representative quotes.

## Results

Participants were candid in their responses to questions in the interview guide and included stories and examples from their own and their peers’ experiences as adolescents. The broad themes and subthemes that emerged from the interviews were grouped under two general topics: adolescent’s partner choices and partner influences. The two themes are described in the following subsections.

### Partner choice

Responses to questions about characteristics adolescent girls look for when choosing a partner varied based on the personal interests of the participating women, and they are reviewed in the following subsections. Table 1 displays subthemes with regard to desirable partner characteristics. We identified four themes promotive of risky behavior: “partner is rebellious,” “partner emotional and financial support,” “wants a boyfriend,” and “partner is older/experienced.” The other four themes focused on positive partner characteristics, such as personality traits or interests in specific activities.

**Table 1:** Desired Partner Characteristics

Subtheme	*n*	Sample Quote
Partner is rebellious	6	“I always looked for the bad boys…the ones that were doing the exact same thing I was…going out and smoking weed and at times, you know when I would drink or when I used to use meth.”
Emotional and financial support	5	“I think that girls want to find a man who can support them and who can um who has a job and who has a good head on their shoulders.”
Wants a boyfriend	5	“Usually girls just want a boyfriend just to have a boyfriend. You know, just for cuz I mean to have someone through high school is awesome…”
Partner is older or experienced	3	“The way that they got to do whatever they wanted. I think, like it seemed like they were a lot more free to do as they pleased. And that was somehow attractive.”
Positive partner characteristics	14	“Somebody who’s, like, popular, and good looking, and has a lotta friends.”

#### Partner is rebellious

The theme of adolescent girls looking for rebellious partners emerged in 6 of the 15 interviews. Respondents identified that, as adolescents, they looked for partners who were “reckless,” “dangerous,” “rebellious,” “trouble,” and who were “bad boys.” The women said that, as adolescents, they looked for partners who used drugs and alcohol and “beat people up.” For example, when one respondent was asked what she thought adolescent girls looked for in a partner, she responded, “Horrible things. I don’t think teen girls have the best decision making when it comes to their partners…I didn’t. I had no standards…I think a lot of teenage girls now look for who’s a little bit rebellious.” Other participants echoed similar opinions, and one woman summed up her feedback by saying, “Me and my friends dated all those types of douchebags.”

One participant said that she liked the rebellious adolescent boys because of the commonalities they shared: “I always looked for the bad boys…the ones that were doing the exact same thing I was…going out and smoking weed and at times, you know when I would drink or when I used to use meth.”

Another participant explained that she had found rebelliousness attractive because of the sense of freedom it brought: “Nobody wants to go to school. General consensus, nobody wants to go to school. So when a boy kind of skips school one day, it shows some of, like, freedom that he has and it might attract you.…” Another participant explained the attraction to rebellious partners a different way, saying, “I read once in a good book is you go for the love you think you deserve.” She went on to explain that “a lot of girls who have had a hard time, who are in foster care, or had bad relationships, go back to those bad relationships. Which is not healthy.” In general, these 6 participants talked candidly about how, in retrospect, they regretted that they had been interested in rebellious partners as adolescents.

#### Partner emotional and financial support

Five respondents mentioned that adolescent girls looked for a partner who could support them, to help compensate for support they lack at home. The women defined this kind of partner support in various ways, including attention, comfort, or advice; help feeling good about themselves; and the potential of financial support. For example, one respondent described the attention she sought as an adolescent in this way: “They [adolescent girls] look for somebody who gives them attention.…Girls like me who have had crazy childhoods, or you know, you know like not the most picture perfect childhoods…but I think [they look for] someone who pays attention to them.” Another respondent talked about how she sought a partner who could give her support, given her past experiences, saying, “I want someone who can relate to me. Who’s had some issues and who can comfort me and give me advice on how they went through things that so that way we can both go through it…” A third respondent talked about financial support in addition to emotional support, saying, “I think that girls want to find a man who can support them and who can … who has a job and who has a good head on their shoulders.”

#### Wants a boyfriend

Five respondents mentioned that adolescent girls just want a boyfriend and seem less concerned with the adolescent boy’s other characteristics. One participant put it simply, saying, “Usually girls want a boyfriend just to have a boyfriend. You know? Just for, cuz, I mean to have someone through high school is awesome…” Another woman talked about how she was shy (“I was always really shy when it came to boys”), and although she wanted a boyfriend (“there’s not like a how-to book to get a boyfriend”), she seemed to care little about who that person was as long as they “make the first move.”

Another participant said that adolescent girls are just looking “to hang out” with male partners because “maybe they’re lonely.” Later she talked about how she thought adolescent girls commit to long-term relationships “…cuz they wanting to grow up because their life was so miserable when they were younger and they wanna have a better life so they’re going to find this first guy that they really love.”

#### Partner is older/experienced

Three participants mentioned that they thought adolescents were into older or more experienced partners. For example, one participant said she looked for “someone that’s been in long-term relationships or long relationships, like longer than a week.” Another participant explained that being older was another sign of the partner’s freedom, saying, “The way that they got to do whatever they wanted. I think, like it seemed like they were a lot more free to do as they pleased. And that was somehow attractive.” She went on to explain that it might also be because of the issues she and her peers faced as adolescents: “Maybe had somethin’ to do with being like some sort of like a father figure. I don’t know. Lots of daddy issues.”

#### Positive partner characteristics

When talking about positive partner characteristics, the descriptions were brief and included less detail than the women’s descriptions of the qualities promotive of risky behavior. Fourteen women mentioned the importance of a potential partner’s social status or attractiveness. One woman’s comment about looking for “somebody who’s, like, popular, and good looking, and has a lotta friends” was representative of the types of things said by the other participants. Seven of the women used individual words or short phrases when identifying positive personality traits that they looked for in a partner such as, “funny,” “humor,” “somebody they can probably share their emotions with or trust,” “someone who is open and communicates,” “reliable,” “has plans and goals for their future,” “kind,” “respectful,” “fun,” “patient,” “honest,” “understanding,” “soft-spoken,” “sweet,” “adventurous,” and “a good friend.” Three women also mentioned that they looked for partners interested in specific positive activities, including “art,” “music,” “outdoors,” “sports,” or “physical activity.”

### Partner influence

Most responses to questions about partner influence on adolescent girls related to drug use, talking about safe sex, and practicing safe sex. Each of these subthemes is reviewed in the following subsections. Table 2 displays themes and subthemes about partner influence on adolescent girls’ drug use, talking about safe sex, and practicing safe sex.

**Table 2:** Partner Influences

Subtheme	*n*	Sample Quote
Theme: Partner Influence on Drug Use		
Fitting in	5	“Like say if I was with someone that was using drugs um, and if they used them around me I would want to feel cool or whatever. Um, and just kind of like fit in with his crowd.”
Pressure	9	“…they make it seem cool or make it seem right, make it to where that’s what they’re supposed to be doing. It’s pressuring.”
A common experience	6	“…my sister had done it here and there, you know, she’d once in a while but she never really did it all the time. And she started dating her, and uh, whenever she uses something, it doesn’t matter if it’s pot, alcohol, what she has to use it when she uses it cuz they say that, she says she don’t want her to be high and her not cuz then they might not communicate the same way.”
Improve or maintain the relationship	4	“I didn’t want them to break up with me. You know I, I oh well if I do this with him, he won’t break up with me.”
Proximity or access	5	“Because they’re around each other and they wanna do what each other are doing.”
Theme: Talking About Safe Sex		
Awkward or uncomfortable	10	“It’s not gonna be like some made out scene like how they say in Sex Ed, because really, in all reality, it’s not. You don’t think, hey we’re gonna have sex in two hours let’s talk about this for an hour and then cuddle for 15 min and then you know, do all this other stuff for you know another 45, and then we could have sex.”
Nervous, scared, or unsure	3	“Neither partner is really actually has developed a close relationship with each other and they’re not actually comfortable enough with each other to talk about things that could be awkward or embarrassing and serious.”
Theme: Practicing Safe Sex		
Not thinking about consequences	10	“…they don’t have a condom but they wanna do it now, so they just do it…”
Don’t like condoms/birth control	7	“Because it feels better [without a condom]. Or they just don’t wanna wear that. Or they’re allergic… The way that it hurts their area or whatever”
Alcohol or drugs	6	I didn’t really take the time to get to know any of em. So it was like extremely uncomfortable. I was always on drugs. You know or alcohol or something. So I didn’t, I really never connected to em. So it made it extremely awkward to talk to anybody.
Partner is dishonest	3	“…and then after we slept together my aunt found out and told me that he had herpes and I didn’t even know because he didn’t tell me. And I got really upset…”
Male pressures female	4	“I was raped one time. Well, a couple of times, but this one stands out the most because I was young. It was with a boyfriend and I had told him no and he forced it on me anyway and he was a lot stronger than I was. And there was no fighting him off.”
Unrealistic expectations	5	“Just because he says he cares about you before doesn’t mean that he still will afterwards. I’ve seen that way too many times…He’ll just leave or that’s all you will do afterwards, when you refuse to, he breaks it off with you. That happened to a very good friend of mine.”
Putting on a condom is awkward	1	“I think sex in itself is a pretty awkward when you’re doing it with somebody you don’t really know, and so to have to put on a condom or to put in a female condom or to do something like that in the middle of sex its already awkward just makes it 10 times more awkward. And then you kinda just don’t want to do it anymore. Or it’s just like you’re embarrassed.”

#### Partner influence on drug use

During the interviews, all 15 women identified that they had used drugs as adolescents. The women identified 6 general reasons an adolescent girl might be more likely to use drugs when her partner uses drugs. These reasons included being accepted or fitting in, pressure, a desire for a common experience, to keep the guy or improve the relationship, the proximity and access to drugs, and curiosity.

##### Fitting in

Five respondents explained that using drugs with a partner is a way to fit in. Participants used words stating that they wanted to be “accepted,” “cool,” and to “fit in with his crowd.” Most participants described wanting to fit in with a simple sentence before moving on to a different idea. For example, one participant simply said, “Maybe just to be accepted…I mean to be cool, I guess…” Another said, “Like say if I was with someone that was using drugs, um, and if they used them around me, I would want to feel cool or whatever. Um, and just kind of like fit in with his crowd.”

##### Pressure

Nine participants talked about how adolescent girls often feel pressure to use drugs when their partner is using. Several participants briefly explained that adolescent girls often felt “peer pressure” from their partners. For example, one participant explained, “Because her partner feels guilty about doing ‘em [drugs] and to justify what he’s doing and make himself feel better about himself…they [the boyfriend] pretty much get their partner to do it [use drugs] with them…” Another participant said, “They make it seem cool or make it seem right, make it to where that’s what they’re supposed to be doing. It’s pressuring.” A few of the women went into more detail about pressure they or their peers felt from partners to use drugs. One participant told a story about how her boyfriend convinced her to use methamphetamine:

I originally started using meth because my boyfriend at the time used to use it and he loved it and my excuse for not wanting to use it was I didn’t wanna go find it. I didn’t know where it was, I didn’t have the money for it. But, at the same time he knew I wanted to use it because it would get me skinny. And I’ve always have had an unhappy weight and it’s always been a weakness of mine and he knew that and so he took advantage of it and he went and got meth. Went and got everything we needed and then brought it to me and so at that point it was really hard for me to say no.

Another participant talked about how a partners’ pressure to use drugs can be related to the cost of drugs and hope for help in paying for them: “I know a first-hand story I just got yesterday of someone who started heroin for the first time and she was with her boyfriend and she had a job and he didn’t. And so what he did is he filled up a syringe of heroin and stuck it in her thigh. And then she’s like, ‘You know why? Because I had a job and he didn’t and now I’m addicted and so now I can buy his shit too.’”

##### A common experience

Nine of the women described how doing drugs together gives a couple something in common and an ability to communicate on the same level. For example, one participant described it this way: “And um you want to bond with each other! [laughs]” She went on to explain in more detail, saying, “…like, of course you don’t wanna be with somebody that’s getting high without you, you know? …cuz if not, they’re just gonna be like high all over the place and you’re gonna be sittin’ there like, whoa!” Although most of these participants explained a similar desire for a common experience, another participant went into more detail with a story about her sister:

My sister had done it [used drugs] here and there, you know. She’d [used drugs] once in a while but she never really did it all the time. And she started dating her [partner], and uh, whenever she uses something, it doesn’t matter if it’s pot, alcohol, what she has to use it when she uses it cuz they say that, she says she don’t want her [partner] to be high and her [the sister] not, cuz then they might not communicate the same way. They’re very, very codependent on each other…

##### Improve or maintain the relationship

Four participants said that they felt adolescent girls used with their partners because of a hope that it would improve their relationship. For example, one participant explained her experience this way: “I didn’t want them to break up with me. You know I, ‘oh well, if I do this with him, he won’t break up with me.’ And that’s what started it all.” Another participant talked about how not using together could lead to problems in the relationship if the partner was using, explaining, “Uh, and maybe the drugs can make the relationship harder and so you, maybe your mind would think, ‘well if I do the drugs with him maybe our relationship will be better.’”

Another participant talked about how her boyfriend seemed to be interested in her only when they were using together:

There was a couple times that, like, the guy that I was doing heroin with, that got me into it, there was times that when we were high together he would…spill out everything, all of his feelings to me. That he was really in love with me and that kinda stuff. And then once he got sober he was the complete opposite. And like, he didn’t wanna look at me, he didn’t wanna sit in the same room as me. There’s a lot of swishy-washy when you do drugs and are trying to have a relationship. And there’s a lot of co-dependent on each other.

Similarly, another participant explained she had used drugs with her partner for a long time, and the relationship ended when she decided to stop using:

She [the partner] was using and I was using with her, and I told her, “You know, I don’t want to do this all the time. I don’t care if I do it, you know, maybe one night a year or something, but I don’t wanna do it all the time because…” I showed her a picture. I said, “This is me at fourteen. I look thirty.” Cuz I was on meth. And it just was horrible. Sunken face, messed up teeth, and I was like, “I don’t wanna look like that ever again. And I don’t wanna lose my mind. And I have permanent heart and lung damage from using and I just, who know.” You know? And I told her, “It’s either my heart or a bag of dope,” and she chose meth. So I left her…

##### Proximity or access

Five participants said it is simply a matter of proximity or access to drugs. Most participants put it directly like this participant, who said, “Because they’re around each other and they wanna do what each other are doing.” Another respondent explained the impact of proximity in terms of doing the same things as the people around her: “I think it’s cuz when you love somebody, it’s just you want to do the same things that they’re doing. If your partner’s being productive I think it encourages you to be more productive yourself. But when they’re doing drugs you’re obviously okay with it so you’re obviously gonna start doing it too.”

#### Talking about safe sex

During the interviews, participants shared experiences and opinions about talking about safe sex. The women overwhelmingly agreed that talking about safe sex for an adolescent can be hard. When participants explained why talking about safe sex can be difficult for adolescents, two subthemes emerged: (a) it is awkward or uncomfortable, and (b) the adolescent is nervous, scared, or unsure about how to talk about safe sex. One participant–interviewer discussion illustrated the challenges faced by adolescent girls when talking with partners about safe sex:

Respondent: I’d say the majority of them talk to their home girls and their best friends.

Interviewer: Okay, so they talk to friends, but do they talk to their partners?

Respondent: Not as probably much as it should be talked about I guess. Unless they’re like, in a serious committed relationship. Then you’re gonna talk to them [the partner]. But if you’ve only been dating this guy, for like, let’s say two or three weeks, or even a month or two, you’re not gonna be like, “Hey dude, I wanna boil this down and we can talk about, you know, well, I don’t wanna get chlamydia and I don’t know if you’ve ever seen but it really is, really bad you know? And I wanna baby someday and I just hope that we could use a condom right now and every time that we have sex and I would really…” No dude! They [adolescent girls] ain’t gonna do that. It’s gonna be like, you know, “Hey, lets fuck, no I’m okay [don’t have an STI], c’mon.” You know? And then they’d [the adolescent girl] be like, “Oh, okay, let’s get this condom.” “No I’m not using that.” “Well you know I feel better if we could use condoms cuz I don’t wanna get pregnant.” “Well then get on birth control.” You know? It’s gonna be, you know, short little. It’s not gonna be like some made out scene like how they say in Sex Ed, because really, in all reality, it’s not. You don’t think, hey we’re gonna have sex in two hours let’s talk about this for an hour and then cuddle for 15 minutes and then you know, do all this other stuff for you know another 45, and then we could have sex.

##### Awkward or uncomfortable

Feeling awkward, embarrassed, or uncomfortable during talks about safe sex was mentioned in 10 interviews. “I know for me,” explained one respondent, “that [safe sex] was a pretty uncomfortable topic.” Most of the women just mentioned that it was uncomfortable, but a few respondents went on to explain why they felt uncomfortable. For example, some women talked about how, as adolescents, they were not very close to their sexual partners, so talking about a serious topic like safe sex was uncomfortable. One participant put it this way: “I feel like some of them [adolescent girls] don’t really have a relationship that is in any way permanent. It’s very temporary. Neither partner is really actually has developed a close relationship with each other and they’re not actually comfortable enough with each other to talk about things that could be awkward or embarrassing and serious.” Another participant gave a similar explanation about why she felt uncomfortable talking about safe sex: “Um, just because uhh, just because, you know, I mean, it wasn’t, it wasn’t a long-term boyfriend.”

##### Nervous, scared, or unsure

Seven of the women explained that they were nervous, scared, or unsure of how to talk to their partner about safe sex. One respondent described the reason for adolescent girls’ nervousness this way: “Uh, well I think that when you’re young, and you have that whole like, uh, you don’t really know a lot about it and you haven’t really done it too much yet so you’re kinda nervous and, and um. Maybe the guy laughs at you, I don’t know.” Other participants said similar things, such as, “Girls are scared the guy might laugh, the guy won’t like her anymore, and are scared of what the guy might say.” “They’re afraid that they’ll feel hurt by the response, or that they will offend the guy by asking about STIs,” and “Some girls don’t know what to say in a conversation.”

One participant said that fear keeps adolescent girls from talking about sex with their partners. When asked what they fear, she said, “Talking about it. Fear of what their partner will do. Um, fear of what their partner will say.” Another participant described adolescent girls fear as “…being scared of that person. Um, [the partner] not liking her anymore, or, you know, not getting attention from that person anymore.”

Although participants said that adolescent girls feel nervous or scared, they also talked about the importance of getting over that nervous feeling. One participant expressed regret that she’s 24 and pregnant with her third child and talked about how uncomfortable she has felt talking to partners about safe sex. She went on to say that adolescent girls need to remember the consequences of unsafe sex when they are nervous to bring it up with their partners:

They [adolescent girls] are gonna get nervous [to talk to their partners about safe sex], yeah. But in all reality they have to be reminded that they’re gonna be the ones who be miserable [if they don’t talk about safe sex]. Yeah, it’s gonna be cool, but in all reality if you’re sitting there taking care of one kid, and this guy’s having sex with your best friend, and you just got pregnant by him and you don’t know what to do, and you don’t want an abortion. You’re gonna be sitting there crying because you’re gonna have to take care of a baby in 9 months. And then your whole life is gonna be ruined. And then you can’t go to college, you know? It just boils everything all crazy like.

#### Practicing safe sex

In addition to discussing the difficulty of talking about safe sex, respondents talked about why practicing safe sex is also difficult for adolescent girls. Nine subthemes related to these barriers, illustrating a wide range of reasons adolescent girls might engage in unsafe sexual behavior.

##### Not thinking about consequences

Ten of the women said that it’s easy not to think about the consequences of unsafe sexual behavior while in the moment. Six of those 10 women mentioned that often they can fail to practice safe sex because they are in a hurry to have sex and do not think about using protection. Participants talked about how often couples are engaged in sexual activity, want to have intercourse, and do not have protection, so go ahead without thought of the future. One participant talked about how adolescents can be impulsive in the moment so they don’t practice safe sex, saying, “They don’t have a condom but they wanna do it now, so they just do it…” Other respondents echoed similar stories about unsafe sexual behavior occurring as a result of being in a hurry. One participant explained how she had been taught since middle school by her mother to carry protection, because sexual partners would typically not think about consequences in the moment:

I carried ‘em [condoms] because I never knew. And I wanted to make sure if I get in a situation I have something, because, you know, my mom always told me when I was younger, “You know men don’t care. They’re gonna just do it. If they want to get their dick wet, they’re gonna do it, so you better carry one just in case and tell him he’s puttin’ it on or you ain’t doin’ it.”

When asked why she thought male partners pressured adolescent girls not to use condoms during sex, the respondent said, “Cuz they’re stupid. Cuz it feels better to have sex without a condom.” Another participant related how she thinks men’s attitudes toward women and impulsivity about sex are linked to men not thinking about the consequences of unsafe sexual behavior, saying, “And all they [her male friends] talk about is, ‘I’m gonna fuck this bitch and I wanna fuck that bitch.’ And, you know, ‘That girl’s so hot.’” When she hears this kind of talk, she said that she often tells her male friends what she thinks about the consequences of their actions:

I tell them straight up… “You know, you think it’s cool, really. But in all reality, do you wanna tell your mom, ‘Hey I got this girl pregnant. Now I have to pay child support’? Because I’m [the male] 15 years old and she’s 14, or vice versa? Dude! You’re fuckin’ 18 or 17 and this girl’s fuckin’ 13? You’re going to fuckin’ jail when you’re 18, you know? You’re going to prison and you’re gonna have to be a sex offender for the rest of your life.”

##### Don’t like condoms or birth control

Seven respondents noted that men do not like to use condoms and many of the men they knew chose not to use condoms. One women said simply, “The guys aren’t [using condoms]. A majority of them don’t want to.” When asked why men did not want to use condoms, one participant was sympathetic to the men, saying, “Because it feels better. Or they just don’t wanna wear that. Or they’re allergic… The way that it hurts their area or whatever…But I wouldn’t wanna wear that either, you know what I mean?”

Another respondent seemed skeptical of men who say they don’t want to use condoms or for their female partners to use birth control. Her skepticism was illustrated in this discussion with the interviewer:

Respondent: Guys refusing to wear protection…or not wanting their girlfriends to be on birth control.

Interviewer: Okay. And do you hear of that happening a lot?

Respondent: I’ve heard of it, yes.

Interviewer: Okay. And do you know maybe some reasons that guys don’t want their girlfriends to wear condoms or use birth control?

Respondent: For the condoms because it feels better without [said sarcastically]. I used quotations; sorry, you didn’t see my hands [laughs]. And the birth control, like hormones and stuff like that, because a lot of the times it can make your hormones go crazy.

Interviewer: Okay. So are the boys concerned about the girl’s hormones? Or?

Respondent: [Nods head yes.] Sometimes. I’ve heard guys say that…I heard my friend’s ex-boyfriend say that to her…because it made her crazy. [Said sarcastically]

##### Alcohol and drugs

Six of the women talked about the prevalence and negative ramifications of drugs and alcohol in their adolescent sexual behavior. Participants said alcohol or drugs obscure judgment—either their own or their partners’. One participant said that being under the influence of drugs or alcohol led her to have sex without ever getting to know or talk with her partner: “I didn’t really take the time to get to know any of ‘em. So it was like extremely uncomfortable. I was always on drugs. You know, or alcohol or something. So I didn’t, I really never connected to ‘em. So it made it extremely awkward to talk to anybody.”

Related to drugs or alcohol increasing sexual risk, one participant said, “It does change your judgment. Alcohol is one of the biggest judgment changers, you know? You know, especially with sexual activities. You know somebody can get you all drunk and then try to take advantage.” Another participant talked about how she “didn’t really take the time to get to know any of ‘em [male sexual partners]. So it was like extremely uncomfortable [to talk to the partner about safe sex]. I was always on drugs.” A third participant described the influence of alcohol on her own unsafe sexual behavior experiences like this: “Well, when I did alcohol, I would black out all the time…But I would get so wasted and I’d puke a lot sometimes. It was all fun and games and when I was dating a guy like, I’d hit on all his friends. Maybe make out with a couple of them. You know? Maybe go a little further and regret it in the morning and not even remember. Yeah, that happened a few times.”

##### Partner is dishonest

Three participants explained that sexual partners can lie about or fail to give partners information about their STI status, which they defined as a barrier to practicing safe sex. One participant said that men will lie about their health, saying, “We don’t need it [a condom], it’s okay. I’ve only been with one other girl…I’m clean.” Two participants talked about instances when a male sexual partner had omitted such health information. One respondent talked about how a friend contracted HIV/AIDS from a male partner who did not disclose his health status, saying, “…I knew a girl who waited until she was 19 years old to have sex and had sex with her boyfriend of a year and a half and he had AIDS and he spread it to her…She even calls me crying, ‘The one time I have sex, I get AIDS.’”

Another respondent talked about a personal experience when she found out that a sexual partner had an STI and didn’t tell her:

I don’t think that the individuals who do have the STD are being honest because I’ve had that incident where I had a family friend who was really, really close to my aunt and he ended up wanting to sleep with me. And I slept with him. And then after we slept together, my aunt found out and told me that he had herpes and I didn’t even know because he didn’t tell me. I got upset that he didn’t tell me and then I found out.

##### Male pressures female

Four participants described the pressure male partners put on adolescent girls to engage in unsafe sexual behavior. Three participants discussed this pressure in broad terms, with one saying, “Like they just pressure you not to [use condoms]…they say it either feels better [without a condom] or they have nothing to worry about [they do not have an STI] or…somethin’. They say whatever they got to [laughs].” The fourth participant talked about a personal experience with forced sex, describing a time when her boyfriend raped her: “I was raped one time. Well, a couple of times, but this one stands out the most because I was young. It was with a boyfriend and I had told him ‘no,’ and he forced it on me anyway. And he was a lot stronger than I was. And there was no fighting him off.”

##### Unrealistic expectations

Five respondents talked about how adolescent girls can have unrealistic expectations about their sexual relationships, such as thinking they are ready to start a family as adolescents or that they would be loved by the partner and the relationship would last. For example, one participant said, “Just because he says he cares about you before doesn’t mean that he still will afterwards. I’ve seen that way too many times…He’ll just leave or that’s all you will do afterwards, when you refuse to, he breaks it off with you. That happened to a very good friend of mine.”

One participant described her own experience getting pregnant at a very young age, saying,

But he seemed really sweet at first. Really kind, really understanding. Everything I looked for. But only being with him for two months, I found out that it was just a façade. He put on a mask pretty much just to snag me, I guess…Basically he was just a really conniving, deceitful person who was trying to get me in bed with him. And being so young, like, I trusted him. I believed everything he said. There was no precaution, there was no taking any caution at all with him. It was just go, go, go pretty much.

##### Putting on a condom is awkward

Though many respondents had mentioned that it is awkward to talk about safe sex, only one participant said it was awkward to use condoms. She talked about her experience this way: “I think sex in itself is a pretty awkward when you’re doing it with somebody you don’t really know. And so to have to put on a condom, or to put in a female condom, or to do something like that in the middle of sex is already awkward. Just makes it 10 times more awkward. And then you kinda just don’t want to do it anymore. Or it’s just like you’re embarrassed.”

## Discussion

The women in the study candidly shared their own and their friends’ experiences from their adolescent years that illustrate the connection between drug and alcohol use and sexual behavior for adolescent girls with maltreatment histories. Many of the women in our study talked about how they did not think about the future when making risky decisions about drug use or sexual behavior as adolescents. In addition, most of the women said they regretted that using drugs or alcohol led to sexual experiences that they would not have wanted to engage in sober.

Many adolescents lack the long-term perspective that contributes to healthy decision making [41]. Recent research has shown that adolescence is marked by dramatic growth in brain activity associated with puberty that peaks in early adulthood [42]. Notably, multiple studies indicate that adolescence is a period during which the brain might be more sensitive to learning through experience than during other developmental periods [42]. Specifically, brain imaging studies have shown that adolescents are less likely to engage in risky behavior if they perceive that there will be a negative consequence for their behavior and, conversely, adolescents are more likely to engage in risky behavior if they deem the outcome to result in a reward [43]. Some researchers argue that although this period of rapid growth and development may be a time when adolescents are particularly vulnerable to engaging in risky behavior (e.g., drug use, unsafe sexual behavior), it can also be an opportunity for positive intervention [44]. This brain development research provides insight into opportunities for intervention with adolescents.

### Implications for practice

The experiences and feelings shared by the women in this study highlight the urgent public health need for at-risk adolescent girls to be empowered with more information and skills related to drug use and safe sex practices [45]. Further, although we did not interview young men, several of the women’s interviews provided insight into things that they wished their male counterparts had known. Based on the information shared in the qualitative interviews and current recommendations for comprehensive sexuality education [25], we propose six key implications for education and intervention programs: (1) adolescent girls could benefit from learning about partner characteristics that are promotive of risky behavior and partner characteristics that increase the potential for healthy relationships, (2) adolescent girls could benefit from receiving information about how romantic or sexual partners can influence their own drug use, (3) adolescent girls could benefit from practice talking about safe sex with partners so that they are more likely to do so with a new partner, (4) adolescent girls could benefit from receiving information about safe sex practices and the risks associated with unsafe sexual behavior, (5) adolescent boys could benefit from receiving information about the risks associated with drug use and unsafe sexual behavior, and (6) implications 1–5 could be integrated into comprehensive sexuality education for adolescents.

#### Partner choice

Data from a diverse national study show that adolescents choose romantic and sexual partners with similar characteristics (race, age, socioeconomic status) and similar interests [46]. Many of the women in this study mentioned that, as adolescents, they often sought relationships that led to risky behavior in an attempt to feel cool, independent, cared about, or free. Many of the women also said that, as adolescents, they sought a partner who would support them emotionally or financially [32]. However, when a young woman relies on the support of a partner to survive (either emotionally or physically), the relationship can easily develop into one that is unhealthy or codependent [47]. Similarly, the desire to have a partner or staying in a relationship to have a partner can be associated with adolescent girls’ increased risk for contraction of sexually transmitted infections [31]. This is particularly true for relationships that include abuse and a power imbalance with the partner [31]. Including a component in intervention programs for at-risk adolescent girls to identify negative or risky partner characteristics (e.g., partner uses drugs or alcohol, partner is controlling) and identify partner characteristics that are promotive of a healthy relationship might reduce adolescent girls’ later drug use or unsafe sexual behavior. Several women talked about positive personality traits or interests in specific activities that they had looked for in a relationship as an adolescent. Intervention programs could help at-risk adolescent girls not only identify negative partner characteristics, but also explicitly define the positive characteristics they seek in a partner. Although identifying risky or positive characteristics is no guarantee that adolescents will find or connect with safe, healthy partners, building their skills in this way could reduce the prevalence of unsafe sexual behavior in this population.

#### Partner influence on drug use

Consistent with findings from prior research [48], the women reported that partners had an influence on their adolescent drug use. The women identified several reasons why they or their peers would use drugs and alcohol with a partner. A potential strategy for intervention programs is to provide at-risk adolescent girls with information and skills about partner influence and associated risks. Because recent brain research shows that adolescents often learn best through experience [42], pairing information with explicit practice about how to respond to partner pressure to use drugs could reduce the prevalence of their drug use [28]. For example, several of the women in the study said that they thought adolescent girls use drugs to try to improve the relationship they have with their using partner. Understanding such influences could help those who develop intervention programs customize the message and associated practice to be relevant to the unique influences faced by this vulnerable population.

#### Talking about safe sex

Women in the sample emphasized that talking with a partner about safe sex practices is awkward or uncomfortable for adolescents. The women’s descriptions of feeling nervous, scared, or unsure about talking about safe sex underscore the need for adolescents to be taught how to have these conversations. In addition, our findings suggest that there is a need for adolescent girls to learn how talking with a partner about safe sex practices directly benefits their own health and future opportunities. Consistent with the UNESCO recommendations for skill-based learning [25], intervention programs could teach adolescent girls practical skills for talking with partners about safe sex practices and provide a relatable rationale. An intervention approach that combines skills practice and information incorporates both experiential learning and risk/benefit evaluation, both of which are effective strategies to promote learning in adolescents [25, 42, 43].

#### Practicing safe sex

Many of the women talked about not using safe sex practices for a variety of reasons, including that putting on a condom can be awkward, they thought providing protection was the male partner’s job, they had trusted their partners when they said they did not have an STI, or they did not feel comfortable insisting on safe sex. Other women talked about direct pressure not to use safe sex practices, including the partner using sexual coercion tactics or refusing to use condoms. As a result of engaging in unsafe sexual behavior, many of the women had stories about early pregnancy and/or contracting STIs. In addition, many of the women’s stories illustrated the link between drug use and unsafe sexual behavior during adolescence. Our findings suggest that intervention programs for at-risk adolescent girls could benefit from including developmentally appropriate and comprehensive sex education that incorporates information about the relationship between drug use and unsafe sexual behavior, strategies for responding to pressure from partners, and the rationale and skills for talking with a partner about the safe sex practices described in the “Talking About Safe Sex” subsection.

#### Interventions for adolescent boys

Although we did not talk directly to men in this study, several themes emerged from the women’s interviews that are applicable for adolescent boys. The women in this study confirmed that, as adolescents, their male partners regularly used drugs and/or alcohol and engaged in a range of sexual coercion tactics that promoted unsafe sexual behavior. As would adolescent girls, adolescent boys would likely benefit from sex education intervention programs that combine information and skills practice, including talking about safe sex, practicing safe sex, recognizing and avoiding sexually coercive behavior, and the relationship between drug use and unsafe sexual behavior. As described previously, recent brain research suggests that females and males learn best through a combination of experiential learning and risk/benefit evaluation [25, 42, 43].

#### Comprehensive sexuality education

Due to the study context and population, the implications discussed in this report focus on support and education for adolescents at heightened risk for health-risking behaviors. In addition, the current UNESCO guidelines for comprehensive sexuality education world-wide emphasize a holistic and developmental approach that includes a wide range of topics and life skills to facilitate healthy choices [25]. These guidelines are consistent with positive youth development research linking safer sexual behavior to other positive psychosocial behaviors [24]. Within the comprehensive sexuality education framework, complex concepts and skills related to gender equity, gender norms, consent, intimate relationships, communication, conflict resolution, self esteem, physical and mental health and wellness, emotional development, and socio-cultural norms are as essential as traditional sexual education topics such as puberty, reproduction, and unsafe sexual practices [25]. Specifically, education and intervention programs could incorporate the suggestions outlined in the paragraphs above within a more comprehensive framework that includes a broad spectrum of life skills for a lasting impact. For girls in particular, it is critical to recognize and address the contextual challenges that may impede girls’ ability to adopt safer sexual behaviors such as gender inequality and gender-based violence. Comprehensive sexual education provides girls with the opportunity to explore how socio-cultural norms about gender and sexuality affect how they experience and navigate their own sexuality, identify barriers to accessing services or practicing protected sex (e.g., gender-based violence), and acquire critical knowledge, skills, and values for negotiating such barriers and pursuing relationships based upon mutual respect and equality.

### Limitations and future directions

Despite the study strengths, several limitations should be noted. First, the sample was relatively homogenous. Study participants were recruited from a community in the Pacific Northwest, a region that is predominantly White. Future qualitative research with a more diverse sample recruited from other regions of the United States could expand and potentially enhance the generalizability of findings. Second, because our study focused on the experiences of young women, the sample included only females. Interviewing male participants with a history of maltreatment could provide additional insights into tailoring intervention programs based on gender. Third, we conducted the qualitative interviews at one time point. Our interviews were designed to ask the women to reflect retrospectively on their experiences as adolescents. Asking adolescent participants to reflect on their experiences and then following up with later interviews in young adulthood could illustrate the development of their thinking about these issues over time. Fourth, our interviews focus on only women with maltreatment histories. It would be beneficial to compare their motivations/decision making processes with women who do not have maltreatment histories. This comparison could provide insight into common influences for adolescents as well as those specific to the study population. Finally, further exploration on other factors that contribute to adolescents’ decision making processes would be beneficial. Factors such as socioeconomic status, education, and other social influences are likely to be relevant influencers.

The qualitative interviews in our study were designed to elucidate factors that influence the decision-making processes for adolescent girls with a maltreatment history in regard to partner selection, drug use, and sexual behavior. Examining these factors can provide interventionists and researchers with valuable information to fill a gap in the literature and better understand the experiences of this vulnerable population.

## Conclusion

This qualitative study substantiates the need for additional support for adolescent girls with histories of maltreatment. Five key targets for intervention emerged from the interviews: First, adolescent girls could benefit from receiving information about partner characteristics promotive of healthy relationships, including how to recognize power dynamics in relationships and how to seek out help and support. Second, adolescent girls could benefit from information about how romantic partners can influence their own drug use. Third, adolescent girls could benefit from instruction on how to discuss safe sex with partners. This includes giving adolescents opportunities to practice through role play. Fourth, adolescent girls could benefit from knowing details about what safe sex is and knowing how to identify their needs and desires. Finally, adolescent boys might also benefit from learning about these same risks and practicing these skills. By gearing intervention programs to focus on these key elements, adolescents with histories of maltreatment will be better equipped to navigate romantic relationships promotive of healthy sexual behavior in the context of a social environment where some youth are engaging in drug use. These intervention targets are consistent with the UNESCO recommendations on the core elements of sexual education programs [25].
